# Contribution of Eastern Mediterranean Region countries to palliative care journals from 1991 to 2020 and its relationship to the development of palliative care

**DOI:** 10.1186/s12904-022-01016-0

**Published:** 2022-07-12

**Authors:** Samy A. Alsirafy, Amneh D. Hassan, Mahmoud Y. Sroor, Ismail Samy, Somaia M. A. Mousa

**Affiliations:** 1 Palliative Medicine Unit, Kasr Al-Ainy Center of Clinical Oncology and Nuclear Medicine, Kasr Al-Ainy Faculty of Medicine, Cairo University, Cairo, Egypt; 2 Palliative Care Unit, Hemato-Oncology Department, King Saud Medical City, Riyadh, Saudi Arabia; 3 Palliative Care Medicine, Oncology Centre, King Faisal Specialist Hospital & Research Centre, Riyadh, Saudi Arabia; 4 Clinical Oncology Department, Kasr Al-Ainy Center of Clinical Oncology and Nuclear Medicine, Kasr Al-Ainy Faculty of Medicine, Cairo University, Cairo, Egypt; 5 Kasr Al-Ainy Faculty of Medicine, Cairo University, Cairo, Egypt; 6 Clinical Pathology Department, Kasr Al-Ainy Faculty of Medicine, Cairo University, Cairo, Egypt

**Keywords:** Eastern mediterranean, Scientific publishing, Palliative Care, Development, Indicators

## Abstract

**Background:**

Palliative care (PC) is in an early stage of development in the Eastern Mediterranean Region (EMR) of the World Health Organization. A metric based on publishing in specialized PC journals may be useful in assessing PC development. This study was conducted to describe the contribution of EMR countries to PC research and to study the relationship between this contribution and the levels of PC development.

**Methods:**

The Scopus database was used to search 21 PC journals (1991–2020) for articles with at least one EMR-affiliated author independently of his/her position in the article. As an indicator, the 3-year average articles per million population per year (AAMY) was calculated. Changes over time were calculated through a regression analysis. The relationship between the AAMY and the level of PC development and opioid consumption were assessed through Mann-Witney test using the worldmap PC development categories as a proxy, and Spearman analysis, respectively.

**Results:**

The number of articles published during the 30-year period was 31,108 of which 402 (1.3%) were EMR-affiliated. There was a steady rise in the AAMY of the EMR (*R*^2^ = 0.894). The number of EMR-affiliated articles increased from 3 in the period 1991–1995 to 191 in 2016–2020. The 2018–2020 AAMY was significantly higher in countries with greater PC development than in those without (median [IQR] = 0.0975 [0.0254–0.1802] and 0.0098 [0–0.0256], *p* = 0.042). Also, it was significantly higher in countries that progressed to a higher level of PC development between 2006 and 2017 (*p* = 0.0159). There was a significant positive correlation between the average opioid consumption for the years 2017–2019 and the AAMY for the same period (*p* = 0.0043).

**Conclusions:**

There is a slow steady progress in the contribution of EMR countries to PC journals, which corresponds to the level of PC development and its progress in the region. A metric based on the contribution to specialized PC journals may be a useful indicator of PC development.

## Introduction

The Eastern Mediterranean Region (EMR) of the World Health Organization (WHO) includes 22 countries in East/North Africa and South/West Asia with different income levels and an estimated population of 735.9 million in 2020, which represented 9.5% of the world population [1–3]. Palliative care (PC) is generally in an early stage of development in the EMR [4, 5]. Since the 1990s, the PC movement has progressed slowly in the EMR, with a few countries reaching a level of generalized PC provision, but not an integration level [5]. From 2006 to 2017 the level of PC development had progressed to a higher level in 13 (59%) of the 22 EMR countries [5, 6].

Several indicators related to the availability of services, health policies, the use of essential PC medicines, education, and training have been used to assess PC development [7]. The global output of PC research is increasing and the use of PC research-related metrics as indicators of PC development has been suggested before [8–12]. Recently, the World Health Organization (WHO) recommended PC research-related indicators to assess PC development in countries [7]. However, being recently introduced, the WHO recommended testing the practicality of such indicators before their use.

Over the last three decades, the number of high-quality peer-reviewed journals specialized in PC has increased, with thousands of articles published in them annually. A PC scientific publishing metric based on these specialized PC journals may be a useful indicator to assess and monitor the development of PC in countries.

The aim of this bibliometric study was to describe the pattern of contribution of EMR countries to specialized PC journals during the last 30 years. Another aim was to assess the relationship between the contribution to specialized PC journals and the level of PC development and opioid consumption in EMR countries.

## Methods

Bibliometric study to describe the contribution of EMR countries to a number of specialized PC journals using the Scopus database.

### Eastern Mediterranean Region countries

The 22 member countries of the WHO Regional Office for the Eastern Mediterranean were included in this study [1] (Table 1).

**Table 1:** Estimated population, income level, palliative care development level and average consumption of drugs across the Eastern Mediterranean countries

**Country**	**Population in millions, 2020** [2]	**Income level, 2020** [3]	**Palliative care development level**			**Average consumption of narcotic drugs (excluding Schedule III preparations & methadone) in DDDs for statistical purposes / million inhabitants / day, 2017–2019 **[13]
			**2006** [6]	**2017** [5]	**Change from 2006 to 2017**^a^	
**Afghanistan**	38.9	Low	1	3a	Progression	2
**Bahrain**	1.7	High	2	3a	Progression	272
**Djibouti**	1	Lower middle	1	1	No change	0
**Egypt**	102.3	Lower middle	3	3a	No change	129
**Iran**	84	Lower middle	2	3a	Progression	35
**Iraq**	40.2	Upper middle	3	1	Regression	0
**Jordan**	10.2	Upper middle	3	3b	Progression	258
**Kuwait**	4.3	High	2	3a	Progression	302
**Lebanon**	6.8	Upper middle	2	3a	Progression	248
**Libya**	6.9	Upper middle	1	3a	Progression	33
**Morocco**	36.9	Lower middle	3	3a	No change	47
**Oman**	5.1	High	2	3b	Progression	80
**Pakistan**	220.9	Lower middle	3	3a	No change	1
**Palestine**	4.8	Lower middle	2	3a	Progression	0
**Qatar**	2.9	High	2	3b	Progression	298
**Saudi Arabia**	34.8	High	3	3b	Progression	382
**Somalia**	15.9	Low	1	1	No change	0
**Sudan**	43.9	Low	2	3a	Progression	8
**Syria**	17.5	Low	1	1	No change	71
**Tunisia**	11.8	Lower middle	2	3a	Progression	142
**United Arab Emirates**	9.9	High	3	2	Regression	166
**Yemen**	29.8	Low	1	1	No change	2

^a^A change from level 3 in 2006 to level 3a in 2017 was considered ‘no change’, while a change to 3b was considered ‘progression’

### Data sources

The population estimates from 1991 to 2020 and the classification of countries according to income were retrieved from the World Bank DataBank [2, 3].

The level of PC development was determined according to the global Mapping Levels of Palliative Care Development in 2006 and 2017 [5, 6]. In 2006 mapping, countries were categorized into four PC development levels, 1 (no known hospice-palliative care activity), 2 (capacity building activity), 3 (localized hospice-palliative care provision) and 4 (hospice-palliative care services are reaching a measure of integration with mainstream service providers) [6]. The 2017 mapping categorized countries into six levels, 1 (no known palliative care activity), 2 (capacity-building palliative care activity), 3a (isolated palliative care provision), 3b (generalized palliative care provision), 4a (palliative care services at a preliminary stage of integration to mainstream health care services) and 4b (palliative care services at an advanced stage of integration to mainstream health care services) [5].

Opioid consumption data were obtained from the Technical Report on Narcotic Drugs of the International Narcotics control Board for the year 2021 [13]. The level of consumption was expressed as the average consumption of narcotic drugs (excluding preparations in Schedule III and methadone) in defined daily doses for statistical purposes per million inhabitants per day for the years 2017 – 2019.

### Hospice and palliative care journals

Google Scholar Metrics was used to identify PC journals listed under the category ‘Health & Medical Sciences: Hospice & Palliative care’. Another seven journals were identified by searching Google Scholar Metrics using the search terms ‘palliative’, ‘palliative care’, ‘palliative medicine’, ‘hospice’ and ‘end-of-life’. The journals indexed in Scopus were included in the current analysis. The final list included 21 titles (Table 2).

**Table 2:** List of 21 hospice and palliative care journals included

**Journal title**	**Scopus Source-ID**	**Scopus Coverage**		**No. of articles**^a^
		Start	End	
*American Journal of Hospice and Palliative Medicine*	27,059	1984	Current	2746
*Annals of Palliative Medicine*	21,100,408,176	2015	Current	551
*BMC Palliative Care*	28,112	2002	Current	927
*BMJ Supportive and Palliative Care*	21,100,322,088	2011	Current	718
*Current Opinion in Supportive and Palliative Care*	12,100,155,640	2007	Current	774
*Death Studies*	13,486	1985	Current	1672
*European Journal of Palliative Care*	4,700,152,772	2006	2018	920
*Indian Journal of Palliative Care*	145,701	2005	Current	866
*International Journal of Palliative Nursing*	28,140	2000	Current	1642
*Journal of Hospice and Palliative Nursing*	4,100,151,711	1999	Current	1013
*Journal of Pain and Palliative Care Pharmacotherapy*	23,060	2002	Current	1240
*Journal of Pain and Symptom Management*	16,790	1986	Current	5586
*Journal of Palliative Care*	15,988	1985	Current	1158
*Journal of Palliative Medicine*	15,989	1998	Current	4719
*Journal of Social Work in End-of-Life and Palliative Care*	4,700,152,787	2005	Current	345
*Médecine Palliative*	4,000,148,111	2004	Current	722
*OMEGA-Journal of Death and Dying*	12,759	1970	Current	992
*Palliative and Supportive Care*	4,000,151,904	2003	Current	1293
*Palliative Medicine*	16,484	1987	Current	2658
*Palliative Medicine in Practice*	21,100,929,724	2018	Current	82
*Progress in Palliative Care*	13,062	2001	Current	484
Total				31,108

^a^During the period 1991 to 2020

International collaboration was defined as the affiliations of authors with more than one country.

### Scopus search strategy

The Scopus database was searched (last updated on 1 February 2022) for the documents published in the 21 selected journals using their Scopus Source ID (Table 2). The year of publication was limited to ‘after 1990’ and ‘before 2021’ and the affiliation country was limited to EMR countries (Afghanistan, Bahrain, Djibouti, Egypt, Iran, Iraq, Jordan, Kuwait, Lebanon, Libyan Arab Jamahiriya, Morocco, Palestine, Oman, Pakistan, Qatar, Saudi Arabia, Somalia, Sudan, Syrian Arab Republic, Tunisia, United Arab Emirates or Yemen). The search excluded the ‘article in press’ publication stage and the ‘editorial, erratum, book and retracted’ document types. The search query was as follows:

**Table:** 

SRCID ( 27059 OR 21100408176 OR 28112 OR 21100322088 OR 12100155640 OR 13486 OR 4700152772 OR 145701 OR 28140 OR 4100151711 OR 23060 OR 16790 OR 15988 OR 15989 OR 4700152787 OR 4000148111 OR 12759 OR 4000151904 OR 16484 OR 21100929724 OR 13062 ) PUBYEAR > 1990 PUBYEAR < 2021 AND ( AFFILCOUNTRY ( Afghanistan OR Bahrain OR Djibouti OR Egypt OR Iran OR Iraq OR Jordan OR Kuwait OR Lebanon OR Libya OR "Libyan Arab Jamahiriya" OR Morocco OR Palestine OR "Occupied Palestinian Territory" OR Oman OR Pakistan OR Qatar OR "Saudi Arabia" OR Somalia OR Sudan OR Syria OR "Syrian Arab Republic" OR Tunisia OR "United Arab Emirates" OR Yemen ) ) AND (EXCLUDE ( PUBSTAGE , "aip" )) AND ( EXCLUDE ( DOCTYPE , "ed" ) OR EXCLUDE ( DOCTYPE , "er" ) OR EXCLUDE ( DOCTYPE , "bk" ) OR EXCLUDE ( DOCTYPE , "tb" ) )	

### Scientific publishing metric

The articles per million population per year (AMY) for individual countries was calculated by dividing the number of articles with authors affiliated to the country (independently of their position in the article) in a year by its population in millions for the same year, as estimated by the World Bank [1]. The AMY was calculated for the World and EMR countries in total using the same principle. The 3-year average AMY (AAMY) for a specific year was calculated by averaging the AMY of that year and those of the year before and after.

### Statistical methods

Statistical analysis was performed using MedCalc® Statistical Software version 20.011 (MedCalc Software Ltd, Ostend, Belgium) and Microsoft Excel for Windows. Regression analysis was used to describe the change in AAMY over time. The F-test was used for hypothesis testing. Mann–Whitney was used to test the significance of difference in AAMY according to PC progression and its development. Spearman’s correlation test was used to test the correlation between the AAMY and opioid consumption. A *p* value < 0.05 was considered significant.

The mapping of the 2018–2020 AAMY in the EMR was performed using the worldmap (version 1.3–6) and RColorBrewer packages for the R Language and Environment for Statistical Computing (version 4.1.1) [14] and the RStudio Integrated Development Environment for R (version 1.4.1106) [15].

## Results

As of 1 February 2022, searching the 21 selected journals for the period 1991–2020 yielded 31,108 documents after excluding editorial, erratum, book, retraction, and in press articles. After limiting the search to EMR countries’ affiliation, the final count was 402 (1.3%) documents.

The characteristics of these 402 EMR-affiliated documents are illustrated in Table 3. Almost half of the retrieved documents were published in three journals, the *Indian Journal of Palliative Care*, *Journal of Pain and Symptom Management*, and *American Journal of Hospice and Palliative Medicine*. Seventy-eight percent of the documents were published during the last 10-year period (2016–2020).

**Table 3:** Characteristics of 402 Eastern Mediterranean Region (EMR)-affiliated documents published in 21 hospice and palliative care journals from 1991 to 2020

	No	%
**Journal**		
*Indian Journal of Palliative Care*	94	23.4
*Journal of Pain and Symptom Management*	45	11.2
*American Journal of Hospice and Palliative Medicine*	43	10.7
*Death Studies*	32	8
*International Journal of Palliative Nursing*	27	6.7
*Journal of Palliative Medicine*	24	6
*OMEGA-Journal of Death and Dying*	22	5.5
*Palliative and Supportive Care*	20	5
*BMC Palliative Care*	14	3.5
*European Journal of Palliative Care*	12	3
*Journal of Hospice and Palliative Nursing*	11	2.7
*Journal of Pain and Palliative Care Pharmacotherapy*	10	2.5
*Journal of Palliative Care*	10	2.5
*Palliative Medicine*	10	2.5
*Progress in Palliative Care*	10	2.5
*Médecine Palliative*	7	1.7
*Annals of Palliative Medicine*	4	1
*BMJ Supportive and Palliative Care*	4	1
*Current Opinion in Supportive and Palliative Care*	3	0.7
**Year of publication**		
2016–2020	191	47.5
2011–2015	123	30.6
2006–2010	57	14.2
2001–2005	22	5.5
1996–2000	6	1.5
1991–1995	3	0.7
**Type of document**		
Article	333	82.8
Review	33	8.2
Other	36	9
**Subject area**^a^		
Medicine	310	77.1
Nursing	167	41.5
Psychology	52	12.9
Arts and Humanities	32	8
Social Sciences	22	5.5
**EMR Countries representation**		
Iran	140	34.8
Egypt	60	14.9
Saudi Arabia	60	14.9
Jordan	48	11.9
Kuwait	33	8.2
Pakistan	30	7.5
Lebanon	27	6.7
United Arab Emirates	17	4.2
Morocco	9	2.2
Qatar	9	2.2
Oman	6	1.5
Iraq	5	1.2
Palestine	5	1.2
Tunisia	5	1.2
Sudan	4	1.0
Syrian Arab Republic	3	0.7
Bahrain	2	0.5
Yemen	2	0.5
Afghanistan	1	0.2
Libyan Arab Jamahiriya	1	0.2
Djibouti	0	0.0
Somalia	0	0.0
**Top 10 represented institutions**		
Cairo University (Egypt)	34	8.5
Kerman University of Medical Sciences (Iran)	26	6.5
King Faisal Specialist Hospital and Research Centre (Saudi Arabia)	25	6.2
Kuwait University (Kuwait)	25	6.2
Tehran University of Medical Sciences (Iran)	24	6.0
Tabriz University of Medical Sciences (Iran)	21	5.2
King Hussein Cancer Center (Jordan)	21	5.2
Shahid Beheshti University of Medical Sciences (Iran)	20	5.0
American University of Beirut (Lebanon)	16	4.0
Jordan University of Science and Technology (Jordan)	11	2.7
**International collaboration**		
None (single EMR country)	230	57.2
Single EMR + non-EMR country(s)	133	33.1
Multiple EMR countries only	29	7.2
Multiple EMR countries + non-EMR country(s)	10	2.5
**Top 5 collaborating non-EMR countries**		
United States	70	17.4
United Kingdom	26	6.5
Canada	20	5.0
India	15	3.7
France	12	3.0

^a^Some documents are listed under more than one subject area

In general, the authors represented more than one country (international collaboration) in 172 (42.8%) documents. The authors represented both EMR and non-EMR countries in 143 (35.6%) documents, while they represented multiple EMR countries in only 39 (9.7%).

The number of EMR-affiliated articles increased from 3 (0.21% of all published articles) in the period 1991–1995 to 191 (2% of all published articles) in 2016–2020.

**Fig. 1:**
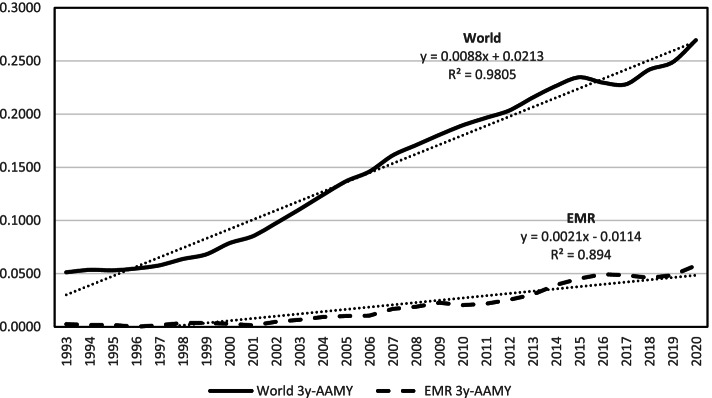
Plot of the 3-year moving average of articles per million population per year (AAMY) for the World and the Eastern Mediterranean Region (EMR)

The trend of the 3-year moving average of AMY for the World and the EMR is plotted in Fig. 1. There was a significant increase in the number of EMR-affiliated articles over the 30-year study period (*R*^2^ = 0.8694, F ratio = 219.3, *p* < 0.0001).

**Fig. 2:**
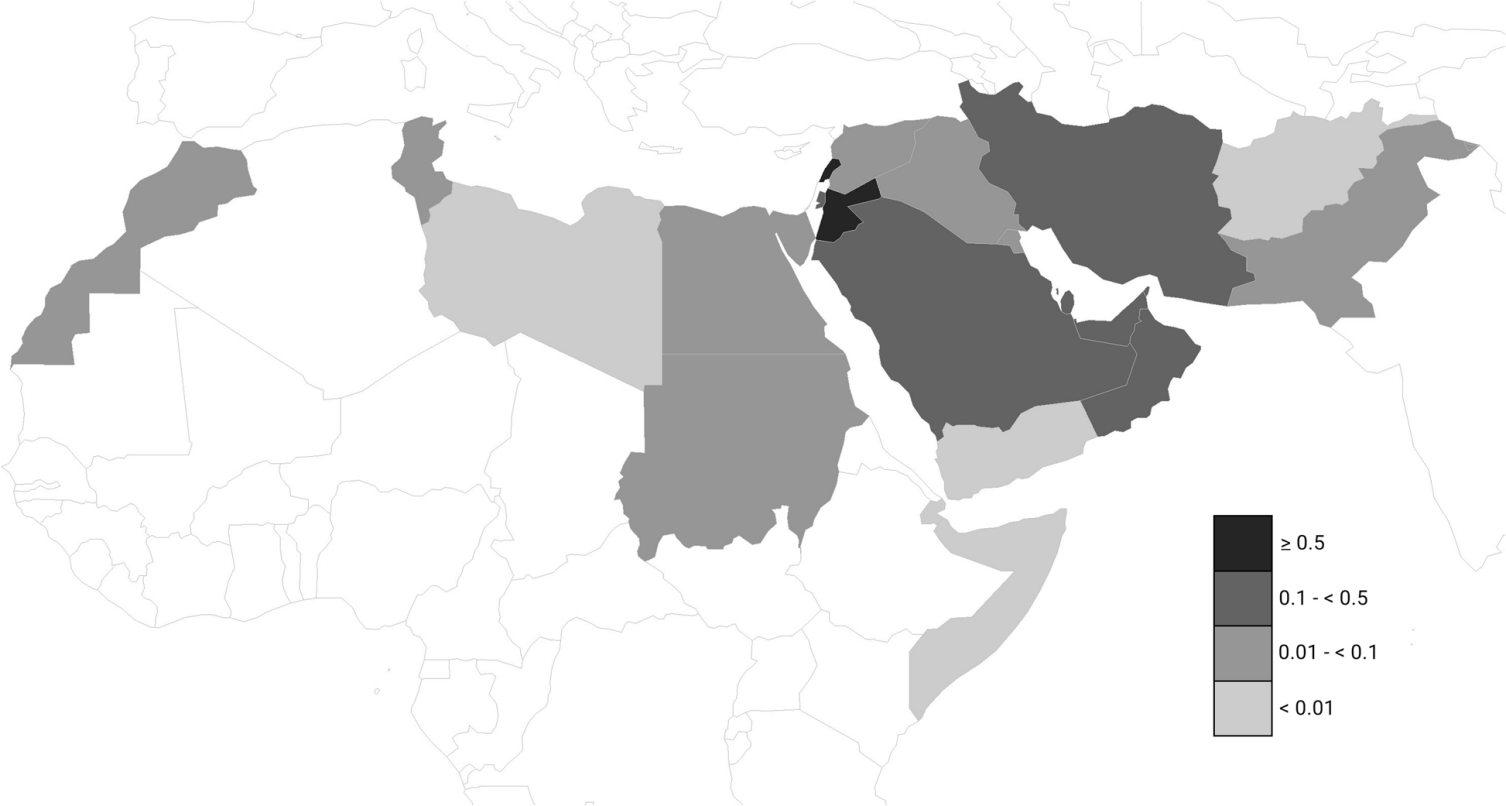
Eastern Mediterranean Region (EMR) map for 2018–2020 3-year average articles per million population per year (AAMY)

The 2018–2020 AAMY for the EMR countries is mapped in Fig. 2. The global 2018–2020 AAMY was 0.2695 and the EMR was 0.0576. The descending order of the EMR countries according to their 2018–2020 AAMY was as follows: Jordan (0.6254), Lebanon (0.5354), Iran (0.2171), Bahrain (0.1959), United Arab Emirates (0.1703), Saudi Arabia (0.1645), Palestine (0.1423), Oman (0.1340), Qatar (0.1157), Kuwait (0.0792), Tunisia (0.0564), Egypt (0.0529), Morocco (0.0274), Iraq (0.0256), Sudan (0.0234). Syria (0.0195), Pakistan (0.0152), Afghanistan (0.0088), Djibouti (0), Libya (0), Somalia (0) and Yemen (0).

Only two EMR countries (Jordan, Lebanon) were above the global average. All Category 3b countries were above the average EMR AAMY and all Category 1 countries were below it.

For comparison, the 2018–2020 AAMY for the top three contributing countries Canada, the United Kingdom, and the United States was 4.2019, 4.0814, and 2.5505, respectively. These values are much higher than those of the EMR.

The 2018–2020 AAMY was significantly higher in countries with palliative care provision (categories 3a and 3b) than in countries without (categories 1 and 2) (median [IQR] = 0.0975 [0.0254 – 0.1802] and 0.0098 [0 – 0.0256], respectively; Mann–Whitney U = 20.5, *p* = 0.042). Similarly, the 2018–2020 AAMY was significantly higher in EMR countries with progression to a higher level of PC development from 2006 to 2017, when compared to countries with no progression (median [IQR] = 0.134 [0.0482 – 0.2012] and 0.0195 [0 – 0.0338], respectively; Mann–Whitney U = 23.5, *p* = 0.0159).

The 2018–2020 AAMY was significantly higher in high- and upper middle-income EMR countries than lower median- and low-income ones (median [IQR] = 0.1492 [0.0792 – 0.1959] and 0.02144 [0.0044 – 0.0547], respectively; Mann–Whitney U = 24.5, *p* = 0.0189).

**Fig. 3:**
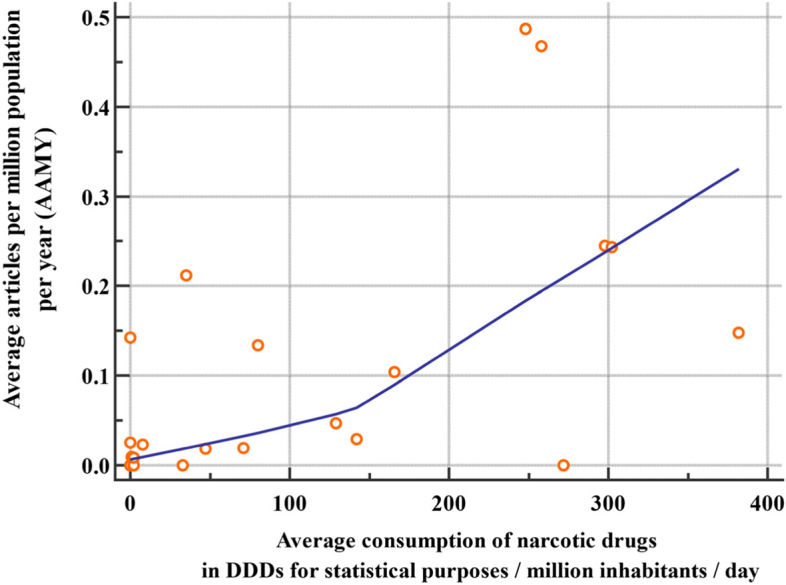
Scatter diagram showing the relationship between the average articles / million population / year (2017 – 2019) and the average consumption of narcotic drugs (excluding Schedule III preparations and methadone) in defined daily doses (DDDs) for statistical purposes / million inhabitants / day (2017 – 2019)

There was a significant positive correlation between the average consumption of opioids for the years 2017–2019 and the AAMY for the same period (Spearman’s rho = 0.584, *p* = 0.0043) (Fig. 3).

## Discussion

This is the first bibliometric study to assess the PC research output of the EMR. Over 30 years, from 1991 to 2020, there was a slow but steady and significant increase in the number of PC publications from the EMR in 21 specialized PC journals. Adjusted for population, the PC research output of individual EMR countries correlated significantly with the level of PC development and opioid consumption.

The results of this study provide an optimistic view of the progress in PC research in the EMR, especially during the last 10 years in which 78% of EMR-affiliated articles were published. However, it should be noted that PC research in the EMR lags behind many other parts of the world, and inequally representing countries. While 10% of the World population reside in the EMR, only 1.3% of the articles in the current analysis are affiliated with EMR countries.

Countries with PC provision and progress in its development had a significantly better PC research performance. As of 2017, the level of PC development in the majority of EMR countries was 3a (localized provision) or 3b (generalized provision) [5]. The AAMY was significantly higher in countries with PC provision, indicating that PC research activity is associated with PC development. This is supported also by the finding that the AAMY was significantly higher in countries where PC delivery is improving, as evidenced by its progression to a higher level of PC development over time.

The main bulk of EMR contribution to PC journals came from three countries, Egypt, Iran, and Saudi Arabia. However, after adjustment for population, Jordan performed best among the EMR countries. Jordan is one of the first countries in the EMR to start PC [16], to recognize palliative medicine as a subspeciality and to accredit a palliative medicine fellowship program [17]. This further supports the close association between PC research activity and the level of PC development.

Opioid consumption is a recognized indicator to assess the development of PC in countries [7]. In the current study there was a significant positive relationship between opioid consumption in EMR countries and their contribution to PC journals. This further supports the possible use of contribution to PC journals as an indicator of PC development in addition to other currently used indicators.

To date, there is no consensus on a PC research-related metric to use as an indicator of PC development. Based on a moderate agreement between international PC experts, the WHO recently recommended the use of the number of peer-reviewed articles as an indicator of PC development in countries [7]. Without adjustment for population, such indicator may give a biased picture of the magnitude of PC research activity in individual countries. For example, 100 publications from a 1 million population country is certainly better performance than 100 publications from a 100 million country. Another limitation to the proposed WHO metric is that it specified few databases (PubMed, CINHAL, and Embase) to search for PC publications. No database is perfectly comprehensive, and consequently many relevant PC publications may be missed. Previous studies assessing PC research output searched databases using common terms referring to PC like ‘palliative care’, ‘hospice’ and ‘terminal care’ [10, 11]. A limitation of this method is that published research relevant to PC may be missed.

The metric that we are suggesting in the current study is based on publications in specialized PC journals. Certainly, such a metric is not comprehensive, and many relevant PC publications are not included. However, the specialized AAMY has the advantage that it includes publications relevant to PC, since they are published in peer-reviewed specialized PC journals. Another advantage is that the specialized AAMY is not based on searching using common keywords such as ‘palliative care’ and ‘hospice’. As mentioned, many relevant PC publications may be missed when the search is based on such keywords only. For example, a study on the pharmacological management of cancer cachexia is relevant for PC, but may not be searchable using commonly used PC search terms. A third advantage is that specialized AAMY does not depend on personal judgement on the relevance of articles to PC.

The association between specialized AAMY and PC development level, its progress and opioid consumption in the current study suggests that a PC research metric based on publications in specialized PC journals may be an alternative to the usual metrics using common PC search terms. Another approach is the combination of both methods, which may yield a better outcome. The encouraging findings of this study warrant further research to investigate the value of specialized AAMY in assessing PC development and progress.

There was considerable international collaboration between the EMR countries and countries outside the EMR (35.6% of the articles), especially high-income countries. On the other hand, the collaboration between EMR countries was limited to only 9.7% of the retrieved articles. This finding is similar to a recent bibliometric analysis of South American PC publications in that the international collaboration between South American countries and those outside of South America was greater than between South American countries [18]. The percentage of articles with international collaboration in South America was less than that in the EMR and was 17.2% for collaboration with non-South American countries and 2.8% for collaboration between South American countries. The figures of international collaboration in PC research from the EMR are encouraging, but there should be more focus on international collaboration between EMR countries (internal EMR collaboration) to address PC issues unique to the region.

## Conclusion

There is a slow steady increase in the PC research from the EMR which corresponds to the slow PC movement in the region as a whole. A metric based on publications in specialized PC journals may be useful in evaluating the development of PC in countries.

## Data Availability

The data used to generate the results of this study are available from the Scopus database (http://www.scopus.com) using the search strategy detailed in the methods section of this manuscript.

## References

[1] World Health Organization Regional Office for the Eastern Mediterranean: Countries. Available from: http://www.emro.who.int/countries.html

[2] The World Bank. 2020. Population estimates and projections. Available from: https://databank.worldbank.org/source/population-estimates-and-projections#. Accessed 22 September 2021

[3] The World Bank. 2020. World Bank Country and Lending groups. Available from: https://datahelpdesk.worldbank.org/knowledgebase/articles/906519-world-bank-country-and-lending-groups. Accessed 15 October 2021.

[4] Osman H, Rihan A, Garralda E, Rhee JY, Pons-Izquierdo JJ, Lima L, Tfayli A, Centeno C (2017). Atlas of Palliative Care in the Eastern Mediterranean Region.

[5] Clark D, Baur N, Clelland D, Garralda E, López-Fidalgo J, Connor S, Centeno C (2020). Mapping levels of palliative care development in 198 countries: the situation in 2017. J Pain Symptom Manage.

[6] Wright M, Wood J, Lynch T, Clark D (2008). Mapping levels of palliative care development: a global view. J Pain Symptom Manage.

[7] World Health Organization (2021). Assessing the development of palliative care worldwide: a set of actionable indicators.

[8] Rhee JY, Garralda E, Torrado C, Blanco S, Ayala I, Namisango E (2017). Publications on palliative care development can be used as an indicator of palliative care development in Africa. J Palliat Med.

[9] Arias N, Garralda E, De Lima L, Rhee JY, Centeno C (2019). Global palliative care and cross-national comparison: how is palliative care development assessed?. J Palliat Med.

[10] Cheong WL, Mohan D, Warren N, Reidpath DD (2019). Palliative care research in the Asia pacific region: a systematic review and bibliometric analysis of peer-reviewed publications. J Palliat Med.

[11] de Lima C, Dos Santos Neto MF, Costa RFA, Franco JO, Calfi GS, Paiva BSR (2021). Characteristics of palliative care publications by South American authors in the last 20 years: systematic literature review with bibliometric analysis. J Pain Symptom Manage.

[12] Sánchez-Cárdenas MA, Garralda E, Arias-Casais NS, Benitez Sastoque ER, Van Steijn D, Moine S, et al. Palliative care integration indicators: an European regional analysis. BMJ Support Palliat Care. 2021; bmjspcare-2021–003181. 10.1136/bmjspcare-2021-003181. 10.1136/bmjspcare-2021-003181 34518283

[13] International Narcotics Control Board (2021). Narcotic Drugs: Estimated World Requirements for 2021; Statistics for 2019.

[14] R Core Team. 2021. R: A language and environment for statistical computing. R Foundation for Statistical Computing, Vienna, Austria. Available from: https://www.R-project.org/.

[15] RStudio Team. 2021. RStudio: Integrated Development Environment for R. RStudio, PBC. Boston. Available from: http://www.rstudio.com/.

[16] Stjernswärd J, Ferris FD, Khleif SN, Jamous W, Treish IM, Milhem M (2007). Jordan palliative care initiative: a WHO demonstration project. J Pain Symptom Manage.

[17] Shamieh O, Richardson K, Abdel-Razeq H, Mansour A, Payne S (2020). Gaining palliative medicine subspecialty recognition and fellowship accreditation in Jordan. J Pain Symptom Manage.

[18] de Lima C, Paiva BSR, Dos Santos Neto MF, Hui D, Perez-Cruz PE, Zimmermann C (2021). The Impact of international research collaborations on the citation metrics and the scientific potential of South American palliative care research: bibliometric analysis. Ann Glob Health.

